# Research Development on Anti-Microbial and Antioxidant Properties of Camel Milk and Its Role as an Anti-Cancer and Anti-Hepatitis Agent

**DOI:** 10.3390/antiox10050788

**Published:** 2021-05-17

**Authors:** Muhammad Zahoor Khan, Jianxin Xiao, Yulin Ma, Jiaying Ma, Shuai Liu, Adnan Khan, Jamal Muhammad Khan, Zhijun Cao

**Affiliations:** 1 State Key Laboratory of Animal Nutrition, Beijing Engineering Technology Research Center of Raw Milk Quality and Safety Control, College of Animal Science and Technology, China Agricultural University, Beijing 100193, China; zahoorcau@cau.edu.cn (M.Z.K.); xiaojianxin-dairy@cau.edu.cn (J.X.); bs20193040395@cau.edu.cn (Y.M.); majiaying@cau.edu.cn (J.M.); liushuaicau@cau.edu.cn (S.L.); 2 Shenzhen Branch, Guangdong Laboratory for Lingnan Modern Agriculture, Genome Analysis Laboratory of the Ministry of Agriculture, Agricultural Genomics Institute at Shenzhen, Chinese Academy of Agricultural Sciences, Shenzhen 518000, China; dr.adnan93@cau.edu.cn; 3 Department of Parasitology, Cholistan University of Veterinary and Animal Sciences, Bahawalpur 63100, Punjab, Pakistan; jamalmkhan@cuvas.edu.pk

**Keywords:** camel milk, lactic acid bacteria, lactoferrin, antioxidant, anti-microbial, anti-cancer, anti-hepatitis

## Abstract

Camel milk is a rich source of vitamin C, lactic acid bacteria (LAB), beta-caseins and milk whey proteins, including lactoferrin, lysozyme, lactoperoxidase, alpha-lactalbumin and immunoglobulin. The lactoferrin plays a key role in several physiological functions, such as conferring antioxidant, anti-microbial and anti-inflammatory functions in cells. Similarly, the camel milk alpha-lactalbumin has shown greater antioxidative activity because of its higher antioxidant amino acid residues. The antioxidant properties of camel milk have also been ascribed to the structural conformation of its beta-caseins. Upon hydrolysis, the beta-caseins lead to some bioactive peptides having antioxidant activities. Consequently, the vitamin C in camel milk has a significant antioxidant effect and can be used as a source of vitamin C when the climate is harsh. Furthermore, the lysozyme and immunoglobulins in camel milk have anti-microbial and immune regulatory properties. The LAB isolated from camel milk have a protective role against both Gram-positive and -negative bacteria. Moreover, the LAB can be used as a probiotic and may restore the oxidative status caused by various pathogenic bacterial infections. Various diseases such as cancer and hepatitis have been associated with oxidative stress. Camel milk could increase antiproliferative effects and regulate antioxidant genes during cancer and hepatitis, hence ameliorating oxidative stress. In the current review, we have illustrated the anti-microbial and antioxidant properties of camel milk in detail. In addition, the anti-cancer and anti-hepatitis properties of camel milk have also been discussed.

## 1. Introduction

There are two distinct species of camel: Dromedary (one-hump camel) and Bactrian (two-hump camel), and the milk from both types of camels is composed of a high level of proteins, minerals and vitamins [1,2]. A total of 2.9 million tons of camel milk production has been recorded annually worldwide [3]. Camel milk is a rich source of vitamin C, lactic acid bacteria (LAB), caseins and whey proteins, such as serum albumin, peptidoglycan recognition protein, alpha-lactalbumin, lactoferrin, lysozymes, lactoperoxidase, immunoglobulin, whey acidic protein (WAP), glycosylation-dependent cell adhesion molecule 1 (GlyCAM-1 and lactophorin, PP3) and milk fat globule proteins [4,5,6,7,8]. The camel milk whey proteins, caseins and LAB have been widely studied for their antioxidant, immune-regulating, anti-inflammatory, probiotic and anti-microbial activities, respectively [9,10,11,12,13]. Besides anti-microbial and probiotic activities, LAB have also been explored for their antioxidant properties [12]. On the other hand, vitamin C in camel milk can prevent excessive free radicals production [14] due to its electron donor ability [15]. It was reported that *Escherichia coli* (*E. coli)* and *Staphylococcus aureus* (*S. aureus)* injection in Waster rats decreased the serum levels of catalase (CAT) and superoxide dismutase (SOD) and increased the oxidative stress biomarker malondialdehyde. In contrast, the supplementation of camel milk enhanced the serum levels of CAT and SOD, but suppressed the levels of MDA in infected Waster rats fed [9].

Although the generation of reactive oxygen species (ROS) is required from the physiological system to maintain the adequate homeostatic balance, consistent exposure to ROS is undesirable. An excessive level of ROS for a long time has been associated with inflammation and diseases such as cancer [16] and hepatitis [17,18]. Ibrahim et al. [15] documented the antioxidant properties of peptides isolated from camel milk whey proteins and caseins. Their findings indicated that these bioactive peptides have substantial radical-scavenging activities, suggesting that they may be used to prevent and treat oxidative stress-related diseases [15]. Other studies have also demonstrated the anti-cancer [19,20] and anti-hepatitis [21] properties of camel milk, which could be ascribed to its antioxidant properties. Thus, in the current review, we have discussed the anti-microbial and antioxidant properties of camel milk and its role as a therapeutic target in cancer and hepatitis.

## 2. The Anti-Microbial and Antioxidant Properties of Camel Milk

### 2.1. The Anti-Microbial Properties of LAB Isolated from Camel Milk

#### 2.1.1. In Vitro Studies on Anti-Microbial Properties of LAB

The anti-microbial activities of LAB isolated from camel milk have been widely studied [22,23,24,25,26]. The genetic characterization of LAB isolated from camel raw milk using 16S rRNA sequencing showed that *Enterococcus* (24.2%), *Lactococcus* (22.4%) and *Pediococcus* (20.7%) were the main genera in raw milk [27]. These genera have a key role against both the Gram-positive and Gram-negative bacteria and protect against multidrug-resistant *Salmonella* [28]. In addition, the LAB isolated from camel milk can be used as probiotics/immunobiotics [29,30], facilitating the host’s metabolism, controlling foodborne pathogens and reducing antibiotic resistance [31,32,33]. Few studies from Iran have reported the anti-pathogenic and probiotic properties of LAB isolated from the milk of one humped camel [34,35]. Two strains (*Enterococcus* and *Weissella*) isolated from camel milk showed probiotic properties by enhancing the metabolism and controlling diarrhea [36]. Other LAB species isolated from camel milk and that have demonstrated probiotic potential include *Bifidobacterium* strains that could be used in functional probiotic food development [37]. Furthermore, the *Lactobacillus acidophilus* *AA105* strain purified from camel raw milk inhibits the growth of other pathogenic bacteria, such as *Staphylococcus* sp., *Bacillus* sp., *Salmonella paratyphi*, *Shigella* sp. and *E. coli* [31]. Besides, several strains isolated from fermented camel milk, such as *Lactobacillus casei* *TN-2* (*L. casei TN-2*), produce bacteriocin, which restrains *E. coli* and *S. aureus* growth [38], while the *E. faecium LCW44* strain showed a potent effect against *Listeria* spp. and *S. aureus* [39], and *L. brevis CM22* against *Listeria* spp. [27]. The potential of LAB species isolated from camel milk are not only antagonistic against pathogenic bacteria such as *E. coli* and *S.* *aureus*, but could also be useful in food poisoning treatments [40]. The problem of antibiotic resistance is causing great uncertainty in the efforts of the world to fight against infectious diseases. LAB such as *Enterococcus faecium S6* have shown anti-microbial activities against *Listeria monocytogenes*, *Salmonella enterica* and *E. coli* [33] and could be considered alternatives to replace antibiotics. It is also worth noting that some of the LAB isolates are more effective than others. Furthermore, it has been reported that *Ligilactobacillus salivarius* (*L. salivarius)* TUCO-L2 isolated from camel milk has stronger probiotic/immunobiotic properties compared to other LAB isolates [29,41]. Understanding the mechanisms by which the different LAB confer their effects will enable researchers to identify the most effective species that can be further developed as anti-microbial agents.

#### 2.1.2. In Vivo Studies on Anti-Microbial Properties of LAB

Quilodrán-Vega et al. [29] demonstrated that *L. salivarius* TUCO-L2 is associated with resistance to pH and a higher concentration of bile salt showed anti-microbial activity against Gram-negative intestinal bacteria. The anti-microbial activity of *L. salivarius* TUCO-L2 was ascribed to its ability to elicit innate immune responses in the intestinal epithelial cells of mice triggered by Toll-like receptor (TLR)-4 activation. It has also been shown that *L. salivarius* TUCO-L2 regulates the chemokines and cytokines mediated by the modulation of the negative regulators of the TLR pathway [29]. Interestingly, oral supplementation of *L. paracasei Pro4* and *L. rhamnosus Pro7* in mice isolated from camel milk mediated the regulation of Toll-like receptor (TLR)-2, interferon (IFN)-g and secretory immunoglobulin A (IgA) to regulate the gut immunity of mice [42]. The immune responses could explain the observed decrease in the number of antibiotics doses and the reduced risk of developing antibiotic resistance in Waster rats [9], anti-schistosomal and anti-pathogenic activity [43] and the protective effect against *S. enteric* in mice [44]. Camel milk can also be used together with antibiotics. A synergic effect between camel milk and ciprofloxacin against *E. coli* and *S. aureus* in rats has been reported [9]. The strong inhibitory effects of exopolysaccharide (EPS) isolated from the *Lactococcus lactis F-mou* strain (LT898177.1) of camel milk have been documented against *S. aureus*, *Pseudomonas aeruginosa*, *E. coli, L. monocytogenes*, *Bacillus cereus*, *Proteus mirabilis, Acinetobacter baumannii, Enterobacter cloacae* and *Candida albicans* [45], and could be used as a natural additive in the food and pharmaceutical industries

### 2.2. Anti-Microbial Properties of Camel Milk Proteins

#### 2.2.1. In Vitro Studies on Anti-Microbial Properties

Camel milk contains lysozymes, lactoperoxidase, immunoglobulins, vitamin C and protective proteins such as lactoferrin and caseins in higher quantities compared to milk from other animals [46] that contribute to the anti-microbial activities of camel milk [47,48]. Lactoferrin is the second major protein after casein found in camel milk [49]. Camel milk lactoferrin has several biological functions, including iron metabolism, promoting immune function and providing defense against pathogens through its bacteriostatic and/or bactericidal properties [50]. This anti-microbial property of camel milk lactoferrin is due to its lactoferricin and lactoferrampin peptides located on the N-terminal tail [51,52]. Camel milk lactoferrin and lactoferrin peptic hydrolysates have especially been implicated in the treatment of typhoid [53] and the growth inhibition of food spoilage bacteria [54], respectively. The anti-microbial activity and synergic effect of camel milk lactoferrin combined with other antibiotics against methicillin-resistant *S. aureus* (MRSA) have been documented [55].

The anti-microbial activities of whey proteins from camel milk have been widely studied [5,56]. These whey proteins have higher anti-microbial activities compared to those of cow milk proteins [57]. It has been shown that camel milk whey proteins significantly inhibited the growth of pathogenic strains of *E. coli* and *L. monocytogenes* [28]. The chimeric peptide derived from camel milk has been documented as an effective anti-microbial agent. Furthermore, it has been reported that chimeric peptide is more effective against oral pathogenic bacteria (*Streptococcus mutans* and *S. salivarius*) compared to 0.2% chlorhexidine mouthwash [58]. A study has shown the anti-microbial and antioxidant properties of camel milk β-casein [59]. Moreover, the caseins in camel milk and their hydrolysates have also been explored for their anti-microbial activities [60,61].

#### 2.2.2. In Vivo Studies on Anti-Microbial Properties of Camel Milk Proteins

In addition to in vitro studies, the anti-microbial properties of camel milk proteins have been explored in different animal models. The anti-microbial properties of camel milk proteins are associated with extracellular nanovesicles (exosomes) in rats [62]. Camel milk proteins prevented *E. coli* and *S. aureus* derived pathogenicity in Wistar rats [63]. In this study, the expression of interleukin-6 and apoptosis-associated genes were down-regulated in rats fed camel milk. The protective role of camel milk was due to its association with modulating the extent of lipid peroxidation in rats [63]. In rabbits, the camel milk lactoferrin significantly enhanced the immunogenicity, creating protection against pathogens [64].

### 2.3. The Antioxidant Properties of Camel Milk

#### 2.3.1. General Description

Reactive oxygen species (ROS), including hydroxyl radicals, nitric oxide radicals, superoxide anions and peroxyl radicals are various kinds of free radicals generated by chemical and biological systems to maintain the adequate cellular homeostatic balance [65]. However, excessive production of free radicals can damage deoxyribonucleic acid (DNA), proteins and lipids and lead to oxidative stress [66]. Usually, these free radicals are destroyed by the natural antioxidant system of the body [67]. If the natural antioxidant system of body is not able to cope with the free radicals properly, a negative chain of reactions is triggered by free radicals in the body, which may lead to the destruction of the cell membrane, block the action of major enzymes, stop the cellular and energy generation processes essential for the proper functioning of the body, prevent normal cell division and destroy DNA [68]. Thus, in the condition where ROS overwhelms antioxidant systems, the protection against oxidative damage is achieved by exogenous supplementation of antioxidants [69]. Camel milk is an excellent exogenous antioxidant supplement that can be used to attenuate the oxidative stress associated with many diseases, such as hepatitis and cancers [70,71,72]. The caseins, LAB, bioactive peptides and whey proteins, especially lactoferrin, are the major constituents of camel milk having the antioxidant properties

#### 2.3.2. The Antioxidant Properties of LAB Isolated from Camel Milk

Besides anti-microbial activities, LAB have been widely studied for their role as antioxidants [73]. Lin et al. reported that LAB protect from the liver injury induced by oxidized oil by upregulating antioxidant genes such as glutathione S-transferase (GSTO1), heme oxygenase-1 (HO-1), glutamate cysteine ligase (GCL) and NAD(P)H; quinone oxidoreductase-l (NQO1) was upregulated in mice liver cells [73]. In addition, the EPS isolated from *L. acidophilus* have antioxidative and biofilm inhibiting properties [45,74]. Guo et al. demonstrated that the EPS inoculation significantly increased the level of CAT, SOD and glutathione peroxidase (GSH-Px) activity, while it decreased MDA levels in the serum and livers of mice. Furthermore, EPS displayed a scavenging activity of superoxide anion, hydroxyl radical and diphenyl-2-picryl hydrazyl (DPPH) [75]. Previous reports have also documented the antioxidant and anti-colorectal cancer effects of LAB [46,76]. A study reported that LAB administration in mice reduced the oxidative stress caused by doxorubicin. Furthermore, it was documented that the LAB restrict free radicals’ excessive production by scavenging and might be a target in diseases associated with oxidative stress [77]. It was documented that LAB enhanced antioxidant activity and mitigated circulatory oxidative stress to protect cells from damage induced by oxidative stress [78]. The LAB prevented oxidative stress damage by producing several antioxidative enzymes such as CAT, SOD, flavin-dependent oxidase and peroxidase [77]. Although LAB have antioxidant properties and relieve oxidative stress, the underlying molecular mechanisms of LAB to prevent oxidative stress are unclear and need to be elucidated in future research.

#### 2.3.3. The Antioxidant Properties of Camel Milk Proteins

The peptides isolated from camel milk have been shown to have a wide range of biological properties, including antioxidant activity and radical scavenging properties. Therefore, a series of investigations have been performed to examine the antioxidant properties of these natural products obtained from camel milk. A study conducted by Homayouni-Tabrizi et al. reported that peptides isolated from camel milk significantly increased the expression of antioxidant genes (CAT and SOD) in treated HepG2 cells. These findings showed the antioxidant and radical scavenging properties of bioactive peptides isolated from camel milk [79]. The free radical scavenging and antioxidant properties of camel milk casein were increased, followed by enzymatic digestion with pepsin and pancreatin [60]. A greater expression of antioxidant genes in the liver cells from rats treated with camel milk was observed. Furthermore, the results showed that the rats treated with carbon tetrachloride (CCL4) followed by administration of camel milk had increased levels of CAT, SOD, glutathione peroxidase (GPx) and glutathione-S transferase in rat liver cells, suggesting that this milk can prevent oxidative stress caused by CCL4 [80]. In rabbits, camel milk normalized the level of free radicals arising from oxidative stress caused by diabetes by increasing antioxidant genes (SOD and CAT) [81]. Furthermore, it has been documented that 5-fluorouracil and methotrexate induced oxidative stress by suppressing the level of antioxidant genes (CAT, SOD, GPx and total antioxidant capacity (TAC)) in rat kidney cells, while camel milk increased the expression of antioxidant genes to prevent kidney injury caused by 5-fluorouracil and methotrexate [82,83]. Camel milk prevents 5-fluorouracil and methotrexate-induced kidney injury and rheumatoid arthritis by regulating antioxidant and anti-apoptotic markers. The 5-fluorouracil and methotrexate, through mitogen-activated protein kinase (MAPK) and nuclear factor-κB (NF-κB) signaling, induced apoptosis and oxidative stress in rats. Camel milk suppresses the MAPK and NF-κB signaling to neutralize oxidative stress and relieves cells from apoptosis [82,83,84].

The possible reason for the anti-cancer properties of camel milk is the presence of protective proteins such as IgG, IgA, lysozyme, lactoperoxidase and lactoferrin [85,86]. Camel milk protein hydrolysates have shown antioxidant properties and their potential for application in functional foods [87]. Ayyash demonstrated that camel milk fermented with probiotic bacteria from camel milk had the greater anti-cancer potential by inhibiting angiotensin-converting-enzyme (ACE), α-amylase and α-glucosidase [19], as well as antidiabetic and antihypertensive potential compared to bovine fermented milk [20]. Similarly, Maqsood reported a higher antioxidative and angiotensin-1 converting enzyme (ACE) inhibitory effect and antihypertensive properties of camel milk proteins compared to cow milk [88].

Leucopenia is caused by cyclophosphamide (CYP), a cytotoxic anti-cancer agent in rats. Camel milk prevented the CYP-induced toxicity, restored the antioxidative status and increased the expression of SOD and CAT in liver homogenates rats with leucopenia [89]. Similarly, the *Lactococcus lactis* subsp*. creemoris* fermented camel milk significantly increased the expression of antioxidant genes such as SOD, CAT, GPx and glutathione (GSH) and vitamin C levels which were depressed by CCL4 in cardiac cells of rats [90]. On the other hand, the CCL4 also increased the levels of cytotoxic agents (alanine aminotransferase (ALT), lactate dehydrogenase (LDH), creatine kinase (CK), creatine kinase MB (CKMB) and Troponin I); however, significant suppression of these parameters was documented post-treatment with *Lactococcus lactis* subsp*. creemoris* fermented camel milk in rats [91]. Furthermore, immunotoxicity and oxidative stress were regulated in albino rats through cyclophosphamide (CTX). In addition, the CTX had significantly down-regulated the expression of antioxidative genes such as CAT, SOD and GPx, thereby enhancing the MDA expression. However, the co-administration of camel milk exosomes normalized the antioxidant status and restored the immunity after severe suppression by cyclophosphamide in albino rats [62]. It has been reported that in patients with diabetes mellitus a marked decrease in antioxidant genes occurred. The diabetic mellitus-induced rats followed by treatment with camel milk significantly increased the levels of SOD, CAT and GSH. Moreover, camel milk speeds up wound healing in diabetic mellitus-induced rats by regulating the immunity and balancing the oxidative state [92]. We have summarized the antioxidant properties of camel milk in Table 1.

## 3. Camel Milk as a Therapeutic Target in Cancer Treatment

It has been widely studied that camel milk has antioxidant potential and may regulate the genes that prevent/decrease the growth of cancer cells or downregulate those that promote their growth. As already mentioned, camel milk contains lactoferrin and immunoglobulins [49]. Lactoferrin is an iron-binding glycoprotein that has been shown to have antitumor activities in vitro and in vivo [102].

**Figure 1:**
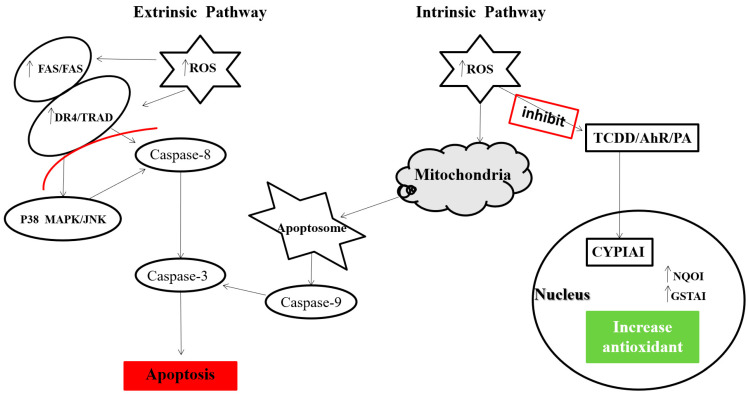
The effect of camel milk as an anti-cancer. Camel milk regulates the apoptosis in cancer cells by using extrinsic signaling to mediate ROS production and mRNA expression of DR4. Further, the ROS and DR4 activate the JNK and caspases to regulate the process of apoptosis. Camel milk also inhibits carcinogenesis via intrinsic pathways by down-regulating the cytochrome P450 1A1 (CYP1A1) and up-regulating the NQO1 and glutathione S-transferase a1 (GSTA1), which provide protection against cancer.

Camel milk lactoferrin prevents the proliferation of colorectal cancer cells and exerts antioxidant and DNA damage-inhibitory properties in cancerous cells [72]. Any abnormal regulation of apoptosis promotes tumor development and metastasis process. Camel milk regulates the apoptotic pathways, thereby stopping the cancer cells’ proliferation [103,104]. Korashy et al. examined the influence of camel milk on human cancer cells’ proliferation, utilizing an in vitro model of the human hepatoma (HepG2) and human breast (MCF7) cancer cells. They argued that camel milk inhibited the proliferation of HepG2 and MCF7 cancer cells by activating the caspase-3 mRNA and activity and the induction of death receptors in HepG2 and MCF7 cell lines [104]. Consequently, the expression of oxidative stress markers, heme oxygenase-1 and ROS production was enhanced by camel milk in HepG2 and MCF7 cell lines [105]. Interestingly, camel milk induced the cell surface death receptors-4 (DR4) mRNA, which is involved in the activation of caspase-3, in mice HepG2 and MCF7 cells [104]. The cell surface DR4 is associated with apoptotic induction [106], which also activates the caspases [107]. The antitumor agents (doxorubicin, cis-platinum and irradiation) used in acute leukemia, multiple myeloma and solid tumor cell lines also upregulate the DR4 mRNA expression [108]. The levels of ROS production and oxidative stress biomarkers were enhanced in the HepG2 and MCF7 cell lines treated with camel milk [105]. Ravagnan et al. [109] documented that mitochondrial ROS production was associated with caspase-3 regulation. The camel milk caseins and whey proteins have been shown to have cytotoxic and antioxidant activities against the MCF7 cells [110]. Camel milk regulates antioxidants and apoptosis through intrinsic and extrinsic pathways, as summarized in Figure 1. Altogether, we conclude that camel milk activates both the extrinsic and intrinsic apoptotic pathways to inhibit the survival and proliferation of HepG2 and MCF7 cells.

Lactoferrin inhibited the activation of Cytochrome P450 1A1 (CYP1A1), which regulates cancer development in a 7,12-dimethylbenz-anthracene (DMBA)-induced hamster buccal pouch carcinoma model. A study with a murine hepatoma Hepa 1c1c7 cell line revealed that camel milk significantly modulates the aryl hydrocarbon receptor-regulated genes such as CYP1A1, NAD (P)H: NQO1 and glutathione S-transferase a1 (GSTA1). Furthermore, it was reported that camel milk suppressed the expression of cancer-inducing gene CYP1A1 and up-regulated the expression of cancer-protecting genes NQO1 and GSTA1 in the murine hepatoma Hepa 1c1c7 cell line at the transcriptional and post-transcriptional levels [111]. The NQO1 and GSTA1 genes also play a key role in reducing several environmental contaminants and endogenous compounds that sustain endogenous antioxidants and shield tissues against cancer-causing agents and oxidative stress damage. Actinomycin D, a ribonucleic acid (RNA) synthesis inhibitor, completely blocked the induction of NQO1 mRNA by camel milk, recommended as a requirement for de novo RNA synthesis through a transcriptional process [111]. Recently, Krishnankutty et al. examined the proliferation, viability and migration of human colorectal HCT 116 and breast MCF-7 cancer cells in response to camel milk. They observed that camel milk also significantly regulates the cytotoxicity in HCT 116 and MCF-7 cells. A decrease in viability, migration and proliferation of HCT 116 and MCF-7 cells was especially observed in response to higher concentrations (100 and 250 μL/mL after 48 h) of camel milk [71]. In a different study, camel milk induced autophagy in HCT 116 and MCF-7 cells, similar to many other anti-cancer agents that facilitate autophagic fluxes in cancerous cells [112].

Lyophilized camel milk, primarily through the instigation of either the intrinsic and extrinsic apoptotic pathways, suppressed BT-474 human breast cancer cell growth and proliferation. The lyophilized camel milk also induced the caspase-3 mRNA, activity level and the initiation of DR4 in BT-474 cells [113]. Furthermore, the regulation of oxidative stress genetic signatures, heme-oxygenase-1 and reactive oxygen species production in BT-474 cells in response to lyophilized camel milk was reported by Hasson and his co-workers [113].

The whey protein in camel milk influences acute myeloid leukemia cells. The whey protein interrupts the connection between PI3 Kinase (PI3K) and B-cell lymphoma 2 (BCL-2) signals and down-regulates their expression to initiate the process of apoptosis in primary acute myeloid leukemia (AML) cells [114]. The higher expression of PI3K and BCL-2 (anti-apoptotic genes) was noticed in AML patients, which increased the survival of AML cells. In addition, the higher expression of PI3K and BCL-2 was associated with chemo-resistance and tumorigenesis [115]. Previous reports have shown that camel whey protein significantly enhances antioxidative stress and helps in the recovery of damaged immune organs by lowering the expression of the anti-apoptotic BCL-2 gene [116,117]. In the same studies, the whey proteins mediated the migration of B and T cells towards the site of antigen recognition in lymphoid organs.

The alpha-lactalbumin (*α*-LA) protein isolated from camel milk has been explored for its important role as an anti-cancer agent, which is due to its ability to bind oleic acid (OA) [118]. Recently, the antitumor effect of OA in tongue squamous cell carcinoma (TSCC) was examined. It was revealed that OA increased apoptotic cells, suppressed cyclinD1 and BCL-2, enhanced the expression of p53 and cleaved caspase-3 [119]. The anti-cancer activity of the OA–*α*-cLA complex has been studied in four human cancer cell lines (Caco-2 colon cancer cells, PC-3 prostate cancer cells, HepG-2 hepatoma cells and Michigan Cancer Foundation-7 (MCF7)). The OA–*α*-cLA complex caused cancer cell death through the induction of apoptosis and cell-cycle arresting, which inhibited the tyrosine kinase (TK) activity of human cancer cells [118,120]. After binding with α-lactalbumin and lactoferrin, OA forms complexes and selectively targets the malignant cells without causing toxicity in normal cells [120].

Consistently, Badawy et al. documented the anti-cancer effect of camel milk and its exosome on in vitro and in vivo MCF7 cells. In brief, the supplementation of camel milk and its exosomes (orally and injection) significantly decreased the progression of breast cancer cells, thereby enhancing apoptosis by increasing the expression of caspase-3 activity and BCL2-associated X protein (Bax) and lowering the expression of the BCL-2 gene. Besides, camel milk and its exosomes inhibited the oxidative stress- (MDA, inducible nitric oxide synthase (iNOS)), inflammation- (interleukin 1B (IL1B), NF-κB), angiogenesis- (VEGF) and metastasis (intercellular adhesion molecule 1 (ICAM-1) and matrix metalloproteinase 9 (MMP-9))-associated genes [121]. The camel milk and its exosomes significantly improved the activities of antioxidant enzymes (SOD, CA, and GPx) in MCF7 cells. Altogether, the inhibitory effect of camel milk and its exosome on cancerous cells is due to the induction of apoptosis and antioxidative effects. It was experimentally proved that cisplatin in combination with camel milk inhibited hepatocarcinogenesis in rats after initiating cancer-inducing diethylnitrosamine, which is again due to the antioxidant effect of camel milk [122].

The proliferation of cells in physiological machinery occurs under various conditions in almost all tissues. Uncontrolled cell division, however, can cause tissue proliferation and even cancer. The prevention of cell proliferation is, therefore, an important therapeutic process in tumor control. The anti-cancer effects of food protein-generated peptides have been extensively investigated [123]. Milk-derived peptides, especially whey proteins, inhibited breast cancer cell line (MDA-MB-231) and nasopharyngeal carcinoma cells. Kamal et al. [124] reported antiproliferative, anti-cancer (cytotoxicity), antidiabetic and anti-inflammatory activities in liver cancer cells treated with hydrolysates of camel milk whey proteins. Similarly, TR35 (whey protein) isolated from camel milk has an anti-cancer ability and inhibited the progression of human carcinoma cells of the esophagus (Eca109) [125]. Yang et al. showed that TR35 inhibited the development of a xenografted tumor and cell proliferation and induced apoptotic activity in mice and Eca109 cells. Transcriptomic and proteomic studies with TR35-treated cells have also been reported. Among the genes studied, those related to apoptosis and necrosis and other pathways in cancer inhibition were identified in TR35-treated cells.

Camel milk has also been found to be effective against fibrosarcoma in a murine model. The anti-cancer drug etoposide (ETP), which was embedded in liposomes isolated from camel milk phospholipids, slowed down tumor growth and increased survival [104]. Likewise, the anti-cancer agent doxorubicin (Dox) or ETP loaded with camel milk phospholipid showed strong anti-cancer activity in a murine model [126]. The phosphatase and tensin homolog (PTEN) gene with anti-cancer efficacy was lower in tumor-induced cells; however, the PTEN gene was found to be higher in phospholipid-embedded doxorubicin-treated cancer cells. Interestingly, both camel urine and milk can also have anti-cancer effects by inhibiting angiogenesis [127]. Based on the above studies, it can be concluded that camel milk proteins can inhibit cancer cell growth via different mechanistic approaches, including apoptosis, antiangiogenesis, cytotoxicity and antioxidant effects. The in vitro studies showed that camel milk could be used to treat breast cancer, liver cancer, leukemia, nasopharyngeal carcinoma and colorectal cancer. However, further in vivo clinical trials for cancer treatment with camel milk are warranted.

## 4. Camel Milk as a Therapeutic Target in Hepatitis Treatment

The hepatoprotective functions and antioxidant activities of camel milk have been studied in rats [128,129,130,131]. Rats were intoxicated with CCL4 to induce hepatotoxicity and oxidative stress followed by treatment with camel milk. A significant normalization in oxidative stress and blood parameters, including serum enzyme activities, were reported with camel milk treatment [128]. Consistently, camel milk offered a hepatoprotective role against gentamicin-induced hepatic damage in rats [131]. Briefly, Al-Asmari et al. demonstrated that gentamicin caused the alteration in the levels of aspartate aminotransferase (AST), alanine aminotransferase (ALT), alkaline phosphatase (ALP) and lactate dehydrogenase (LDH) in rat serum. Furthermore, MDA, myeloperoxidase (MPO), SOD and glutathione S-transferase (GST) functions decreased significantly. However, the blood biomarkers tested were successfully restored to their normal levels in rats treated with camel milk. Similarly, another study found the hepatoprotective effect of camel milk against thioacetamide, which causes liver cell damage in rats [132].

The *L. paracasei* subsp. *paracasei* strain isolated from camel milk significantly protected the liver cells against injury from lipopolysaccharide (LPS)/D-galactosamine (D-GalN) in rats. Furthermore, the level of Th1-type cytokine IFN-gamma in the camel milk-drinking group was significantly higher than that in the non-drinking camel milk group; however, the level of Th2-type cytokines IL-4 in the group drinking camel milk was significantly lower than that in the non-drinking camel milk group. Camel milk regulates the expression of Th1/Th2-type cytokines. It corrects the imbalance of the Th1/Th2 cytokine network, which could strengthen the cellular immune responses and inhibit the replication of virus DNA, and promote the recovery of chronic hepatitis B patients [133]. Similarly, the serum from HCV patients was tested pre- and post-supplementation of the camel milk. A significant decrease in the level of HCV parameters, including liver function (ALT and AST), and improvement in general fatigue was observed [134]. Camel milk improved the inhibition of HCV-related biological factors, such as pro-inflammatory markers, tumor necrosis factor-a, monocyte chemotactic protein-1, AST and ALT, while enhancing the level of the serum albumin, the anti-apoptotic protein BCL-2, antioxidant status, interleukin-10 and vitamin D [135,136]. Another study reported the antiviral activity of lactoferrin and the naïve polyclonal IgGs isolated from camel milk that inhibited HCV infectivity in Huh7.5 hepatoma. The camel milk IgGs have the potential to identify HCV peptides with significant titers (12 × 103) compared to human IgG. On the other hand, camel milk lactoferrin potentially inhibits the intracellular HCV progression at concentrations of 0.25–1.25 mg/mL [48]. Native camel milk lactoferrin and recombinant lactoferrin and their N and C fragments in Huh7.5 cells might also have antiviral and anti-HCV effects [137,138]. In addition, the inhibitory effect of camel milk lactoferrin on HCV infection has been observed in human peripheral blood mononuclear cells [139] and HepG2 cells [140,141]. One of the most relevant reasons for this anti-HCV activity might be the utilization of heparan sulfate on the human cell surface by lactoferrin, which is also targeted by several viruses, including the HCV virus, for attachment and cell entry [142,143]. Consistent with lactoferrin, camel milk casein showed apoptotic potential and anti-HCV activity in human hepatoma and HeLa cell lines [70].

Alcoholic liver disease (ALD), characterized by hepatic inflammation, increased oxidative stress and microbial imbalances, is another serious disease prevalent in the human population [144,145]. Recently, a study reported that camel milk had hepatoprotective potential and could prevent ALD in mice [146]. Furthermore, Ming et al. demonstrated that camel milk enhanced *Lactobacillus* production in the intestine and down-regulated the expression of inflammation-associated genes, such as interleukin 1 beta (IL-1β), chemokine (C-X-C motif) ligand 1 (CXCL1), interleukin (IL)-17 and tumor necrosis factor (TNF-α) pathways. This implies that camel milk modulates inflammation of the liver and improves intestinal microbial homeostasis, which is induced by acute alcohol injury. In addition, it has been documented that camel milk can ameliorate alcoholic liver injury in rats, which is mediated through the antioxidant, anti-apoptotic and anti-inflammatory mechanisms of camel milk [147]. Based on the published data, it has been concluded that camel milk could treat specifically hepatitis induced by virus. In addition, camel milk protects the liver from the oxidative stress and inflammation caused by chemicals and alcohol.

## 5. Conclusions

Based on our review, we have concluded that camel milk has anti-microbial and antioxidant properties. These properties are due to the presence of caseins, LAB and whey proteins in camel milk. Furthermore, camel milk has an antiproliferative effect in human hepatoma HepG2 cells, human breast cancer MCF7 cells, human colorectal HCT 116 cells and esophageal carcinoma cells (Eca109). The antioxidant and anti-microbial properties of camel milk reduce inflammation in the liver cells and improve its associated health functions. Consistently, oxidative stress is also associated with cancer and hepatitis, while camel milk significantly regulates the antioxidant-associated genetic markers to cope with excessive oxidative stress. Thus, camel milk may be a good add-on against breast cancer, liver cancer, human colorectal cancer and hepatitis to relieve the oxidative stress. In addition, camel milk also regulates gut immunity and metabolic activities and prevents diarrhea due to the presence of LAB. Keeping in view the antimicrobial, antidiarrheal and antioxidant properties of camel milk, it is proposed to conduct further research on the utilization of camel milk in the treatment of other diseases as well.

## Figures and Tables

**Table 1:** Antioxidant properties of camel milk.

Agent	Properties	Experimental Animal	Authors
Camel milk isolated peptides	Antioxidative	Camel	[93]
Camel milk isolated peptides	Antioxidative properties, increase CAT and SOD gene expression	HepG2 cells (Human)	[79]
Camel milk	Antioxidative properties	Rats	[94]
Camel milk	Prevent oxidative damage, increase expression of antioxidant genes catalase, GPx and SOD	Rats	[95]
Camel milk	Prevent CCL4-induced liver damage, increase antioxidant activity	Rats	[96]
Camel milk protein hydrolysates	Antioxidative properties, increase CAT and SOD gene expression	Rats	[97]
Camel milk	Antioxidant and anti-apoptotic properties	Rats	[64]
Camel milk	Antioxidant and decrease oxidative stress markers (malondialdehyde, myeloperoxidase and total antioxidant capacity) in lung tissue	Rats	[98]
Fermented camel milk	Prevent CCL4-induced oxidative stress and increase antioxidant activity in liver	Rats	[99]
Fermented camel milk	Prevent CCL4-induced oxidative stress and increase antioxidant activity in kidney	Rats	[90]
Camel milk	Antioxidant activity, prevent the damage caused by CCL4	Rats	[100]
Camel milk	Enhance the antioxidant gene expression	Rats	[101]
